# Light-Emitting Devices with Conjugated Polymers

**DOI:** 10.3390/ijms12031575

**Published:** 2011-03-01

**Authors:** Deng Xian Yu

**Affiliations:** Research Center for Advanced Functional Materials and Devices, Department of Materials Science and Engineering, Harbin Institute of Technology, Shenzhen Graduate School, Shenzhen 518055, China; E-Mail: xydeng@hitsz.edu.cn; Tel.:+86-755-26033211; Fax: +86-755-26033504

**Keywords:** conjugated polymer, light-emission, PLED, flat-panel display

## Abstract

This article introduces a previous study and tremendous progress in basic theoretical modeling, material developments and device engineering for polymer light-emitting devices (PLEDs).

## 1. Introduction

Organic semiconductive thin films have caused extensive interest among scientists worldwide over the past two decades because of their versatility and ease of preparation, leading to various potential applications in electrical and electronic devices such as transistors, photodiodes and light-emitting diodes (LEDs). In this respect, research using organic materials as the active semiconductors in LEDs has resulted in rapid progress owing to their application in flat panel displays, and the performance of the prototype devices has reached levels for realistic application that could lead to a large scale commercialization in the near future.

Electroluminescence (EL), an electrically driven radiative emission process, is a phenomenon that exists in a wide range of conventional semiconductors, and organic EL was first reported and extensively studied in the 1960s [1,2]. In these early studies, the combination of the injected electrons from one electrode and holes from the other electrode, were identified to play an essential role in the light emission process. In 1987, by using two-layer organic light-emitting diodes (OLEDs), a team in Kodak demonstrated devices with appropriately low operation voltages and attractive EL efficiencies by exploiting suitable small-molecule materials and structures [3,4]. Shortly afterwards, in 1990 a Cambridge research team reported the polymer-based light-emitting diodes (PLEDs) [5,6]. Since then, increasing interest and research activities have developed in this new field, and an enormous progress has been made in the improvement of luminance efficiency, color properties and device reliability by a wide range of studies in the design and synthesis of materials, device fabrications, and physics in these materials and devices.

**Figure 1.**
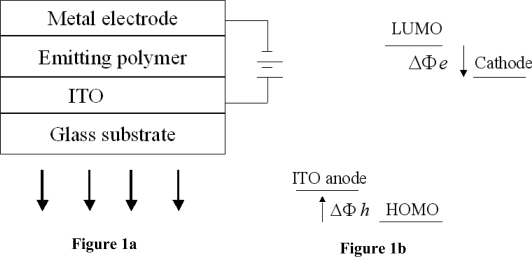
The basic structure (**a**) and the energy level for the operation (**b**) of a PLED.

A PLED consists of very thin layers of polymer films sandwiched by two electrodes. In a typical PLED structure, there are two polymer layers, one of which functions as the hole transporting layer and the other functions as the light-emission layer. An indium tin oxide (ITO) layer is generally used as the transparent anode, which allows the light generated within the diode to leave the device. The metal cathode is conveniently deposited on top of the polymer by thermal evaporation. The basic structure is also illustrated in Figure 1a.

Once a positive electrical potential with respect to the cathode is applied on the anode, holes are injected from the anode to the hole-transporting layer (HTL), while electrons are injected from the cathode. The injected holes and electrons respectively move toward the opposite electrodes, and, within the region of the emissive polymer layer, the recombination of the holes and electrons occurs. Thus, the released energy of the recombination can result in light emission. In the basic structure shown in Figure 1a, ITO has a relatively high work function that is near the highest occupied molecular orbital (HOMO) of the polymers, and it is therefore suitable for use as a hole-injecting electrode, while the low-work-function metals, such as Al, Ba or Ca, are suitable for electron injection. The energy level for the operation of a PLED is shown schematically in Figure 1b.

The EL quantum efficiency of PLEDs is characterized by either the external quantum efficiency (*η*_ext_) or the internal quantum efficiency (*η*_int_), and the former is generally much lower than the latter because the light generated by the excitons cannot be fully coupled through the device. The internal quantum efficiency *η*_int_ is defined as the ratio of the number of photons produced within the device to the number of electrons flowing in the external circuit, which is given by [7]:

(1) $$\eta_{\text{int}}=\gamma r_{\text{st}}q$$

where *γ* is the ratio of the number of exciton formation events within the device to the number of electrons flowing in the external circuit; *r*_st_ is the fraction of excitons which are formed as singlets, since the spin-allowed radiative emission (fluorescene) is only from the singlet, and the triplet excitons do not produce light emission except by some indirect processes, such as triplet-triplet annihilation or phosphorescence; *q* is the efficiency of radiative decay, which depends on the device structure; in particular it is strongly affected by the photonic structure of the device, such as the proximity of the metallic mirrors.

Therefore, to achieve high efficiency PLEDs, several factors need to be considered. They include the balance of electrons and holes, strong radiative transitions for singlet excitons, and efficient light extraction. On the other hand, development of phosphorescence emitters by triplet-triplet energy transfer is also an important method to achieve high efficiency luminescence.

With respect to the function of the polymer used in the PLED device structure, there are three kinds of polymeric materials: light-emission polymer, hole-transporting polymer and electron-transporting polymer. To achieve the balance of holes and electrons within the device, a combination of two or more of the selected function polymers is generally used to attain high luminance efficiency.

A polymer that can be used as light-emission material must have two basic characteristics: electrical conductivity (semiconductive polymer) and high photoluminescence (PL) efficiency. A conjugated polymer has an energy gap between the π (bonding) and π* (antibonding) states, and produces luminescence with an energy level below this energy gap. The corresponding spectrum for EL is similar to that produced by photoexcitation. For example, the energy gap of poly(1,4-phenylene)(PPV) is about 2.5 eV and it produces a yellow-green luminescence [8]. One notes that the spectrum broadens due to vibronic coupling, as is the case for optical transitions in molecular structures. Thus, it is described as an excitonic emission.

Recently, very rapid progress has been made in the research of polymers used for light emission. The emission colors of these polymers have covered the entire visible spectrum, and it is achieved by the structural variety of single-polymers and copolymers. The improvement to these molecular structures plays a very important role for both the luminance efficiency and the stability of the device. These materials were highlighted in the series of PPV, poly (9,9-dialkyfluorene) (PFO), polycarbazole (PCB) and their derivatives and copolymers [9–22]. The synthetic design of the conjugated polymers, in particular the importance of controlling molecular weight, polydispersity and the suppression of defects, has recently been reviewed in detail by Holmes *et al.* [23].

The hole transporting polymer is usually a polystyrenesulphonic acid doped poly(dioxyethylene thienylene) (PEDOT:PSS). This polymer can be spin-coated on a cleaned ITO substrate. It has two major roles, one of which is the formation of a thin film by spin-coating that can smooth the surface of ITO, and the other more important role is that this film has a higher work-function than an ITO film, which is good for hole injection due to the lower height of the injection barrier [24]. By using the PEDOT:PSS as the hole transporting layer, efficient luminance can be achieved in PLEDs that employ many different kinds of polymer materials, especially for red and green-emitting polymers. However, the efficiency is still not perfect for blue-emitting devices; the main reason being that the work-function of PEDOT:PSS does not provide a good match to the energy of the blue-emitting polymers due to the lower level of the HOMO in these wide band gap polymers. An alternative polymer, poly (9-vinyl carbazole) (PVK), was introduced as the hole-transporting layer, which shows a large improvement in efficiency for blue PLEDs [25,26]. However, PVK is a non-conjugated polymer and produces a high electrical resistance within the device, so that the PLED generally shows a high operating voltage. As reported recently, water/alcohol soluble and crosslinked conjugated polymers are preferred for use in multi-layer structures, so these are mostly employed for the hole transport layer [27–29].

An electron-transporting polymer can be blended-in or deposited on the light emitting polymer layer so that the electron transporting properties can be improved. Because the mobility of hole transport is usually much larger than that of the electron transport in a light-emitting conjugated polymer, in order to achieve charge balance, the polymer used as the electron transporter should be added to improve the electron conductivity within the device. Some reports show that the EL efficiency can be enhanced by blending certain polymers, due to their enhanced mobility for electron transport [30,31]. In a multi-layer structure, however, since the electron mobility is generally smaller with respect to the hole mobility for most polymers—especially for the water or alcohol soluble polymers that are good for multi-polymer-layer structure—these polymers are widely used as the hole or exciton blocking layer [32].

**Figure 2.**
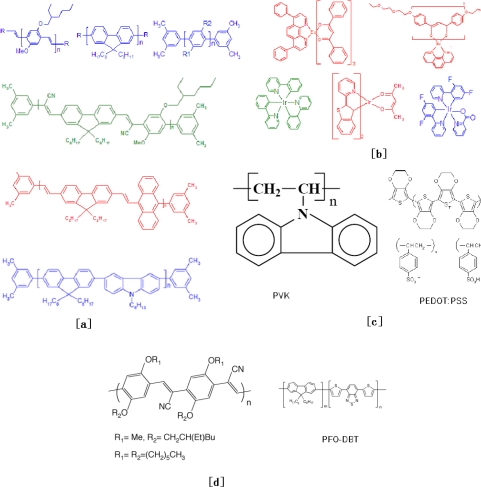
Chemical structures of typical polymers used in a PLED: (**a**) Light-emission polymers; (**b**) Fluorescence and phosphorescence doping molecules; (**c**) Hole transporting polymers; (**d**) [d]Electron transporting polymers.

Figure 2 shows some typical polymers that are used in PLED structures as the light-emission, hole-transporting and electron-transporting layer.

## 2. Charge Injection and Interfaces between the Polymer and the Electrodes

For charge injection at the interface, the charges are required to surmount or tunnel through an energy barrier. The injection ability of the charge is intensively dependent on the characteristics of the interface, so that the interface between the polymer and the electrodes play a very important role for the performance of the device, such as the efficiency and operation voltage. The formation of the interface involves both complicated physical and chemical processes, and the alignment of energy leads at the interface is the most important factor for understanding the charge injection.

In an ideal physical process in which the chemical reactions at the interface are ignored, the energy diagram can be represented as shown in Figure 1b. Here Δ*Φ*_h_ and Δ*Φ*_e_ respectively define the energy barriers for holes and electrons by the Mott-Schottky rule of vacuum level alignment [33]. Although this picture is simple and ideal for the interfacial effect on charge injection, it can nevertheless be successfully used to evaluate the metal electrode’s work functions and the positions of the HOMO and the LUMO of the polymer. ITO provides a relatively good match for hole injection, while low-work-function reactive metals, such as calcium or barium, are generally good for electron injection.

ITO is a favorite choice of anode material. However, although ITO has a good optical transparency, it is not easy to control. Several reports have shown that certain p-doped conjugated polymers are suitable to use as electrodes [34–36]. These doped polymer electrodes have high work functions, thereby they provide low barriers for hole injection to the semiconductor polymer layer. They also provide a good morphology at the interface.

The chemistry occurring at the interface depends on the nature of the metals, the polymers, and especially on the cleanliness of both materials employed, and the vacuum system used in the metallization process. Aluminum and calcium are two typical electron injection metals.

By inserting an ultrathin insulating layer of alkali metal fluorides, such as LiF, between the Al cathode and the emissive layer, it has been shown that dramatically improved electron injection and increased quantum efficiency resulted, both in organic light-emitting diodes (OLEDs) [37] as well as in PLEDs [38–41]. In other approaches, soluble metal ionic polymers, surfactants or metal organic compounds were spin-covered on [42,43], or blended into [44] the emissive polymer to modify the interface between the emissive polymer and the Al cathode, resulting in the enhancement of electron injection and achieved high efficiency PLEDs. The device efficiency of PLEDs using Al electrode is also significantly enhanced by blending or inserting a neutral polymer without any metallic atom or ion [45–48]. It has recently often been reported that water/alcohol soluble conjugated polymers can result in greatly enhanced PLED performance, due to the improvement of the charge injection from high work-function metal cathodes [49–54]. Many relevant physical and chemical processes may take place at these interfaces, but the mechanism for the performance improvement is complex and there has not been a conclusive explanation. When these materials are introduced, good efficiency can be obtained by the device using a high-work-function metal, such as Al. This is a major benefit for the manufacturing process since the reactive metal is not convenient to use for deposition, and the reaction between the reactive metal and the water/oxygen that are present in an environment is generally believed to be the key factor for the stability of the device.

The process of charge injection is also complex. The work-function of the electrode metal, chemical reaction at the organic/electrode interface and the transport of the charges all play a role in the charge injection process. The most important models describing charge injection into a dielectric medium is the Fowler-Nordheim (FN) model for tunneling injection and the Richardson-Schottky (RS) model for thermionic emission [55].

## 3. Electrical Conductivity and Charge Transport in Conjugated Polymers

Within the bulk of the conjugated polymer film, the electrical conductivity is determined by the ability of the charge carriers to move across the film. Generally, the polymer chain lengths are finite in the solid-state film, and in order to move through the whole polymer, the charge carriers have to move between different chains. The transport can then be described as an electron transfer from a charged part of the chain to an adjacent neutral part of the chain. On a microscopic scale, it is one of the major parameters governing the charge transport properties. The ease of transferring charge between adjacent polymer chains is very sensitive to the relative organization of the chains. So the electrical conduction in conjugated polymers is strongly related to the physical arrangement of the molecules in the solid state. This arrangement includes both the position and the orientation of the molecules relative to each other. Also, the rotations, variations of molecular length and angle of backbone bonds in polymers would induce the variation in chain conformation.

The charge transport can be divided into two mechanisms: band transport [56] and hopping transport [57,58], corresponding to the charge transport within and between polymer chains. In both cases, the phonon (vibration of atom) plays an important role. In highly doped conjugated polymers, the partially filled bands are formed and the charges can move along the chains freely except for the scattering by impurities and phonons. After scattering, the direction of propagation of the charge carrier becomes random. Thus, in-band transport phonons are the source of resistance and the conductivity decreases with increasing temperature because more phonon scattering can occur.

In the hopping mechanism, charge carriers move between localized states in the energy band gap. Repeated hopping through different chains leads to the possibility of the charge carriers traveling through the whole sample. During the hopping process, the charge carrier needs to gain external energy for transition from one state to another state. At finite temperatures, electrons can get the external energy from phonons. Phonons are now in this case a source of electrical conductivity, and hopping conductivity increases with temperature because of more available phonons.

## 4. Charge Carriers Recombination and Light Emission

The process of electron-hole capture in these devices is crucial to device operation. Since, in conjugated polymer film, the holes show much higher mobility than the electrons, in order to get efficient capture in the emitting polymer layer, it is necessary to enhance the ability of the electron injection from the cathode. On the other hand, for an effective recombination, the electrons and the holes are restricted to near the interface of the heterojunction by the heterostructures [59,60]. The basic modeling of the charge recombination that is widely exploited is the Langevin theory [61–64].

**Figure 3.**
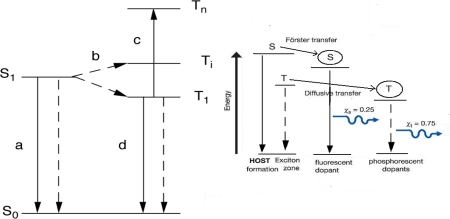
The energy scheme of the two excitonic states (**a**) and the energy transfer process (**b**).

There are two types of excitons that are produced by the electron and hole capture: singlet and triplet. The energy scheme of the two excitonic states is shown in Figure 3a.

The emission from the singlet exciton is called fluorescence. The radiative efficiency of the fluorescence is conveniently measured by photoluminescence. The luminescence efficiency in the solid state tends to be lower than that measured for isolated molecules. This is due both to exciton migration to quenching sites and to interchain interactions that produce lower-energy excited states that are not strongly radiatively coupled to the ground state [65]. By doping with certain kinds of polymer, for example, nonconjugated polymer or wide band gap conjugated polymer, the interchain interaction can be suppressed and the luminescence can be modified. An excitonic energy transfer takes place from one polymer to another polymer with a lower excitonic energy by the Förster energy transfer process. If the latter has more efficient radiative emission it would lead to the high luminescence efficiency [66,67]. The Förster energy transfer process is shown in Figure 3b.

According to the principle of spin statistics, excitons will be formed with spin wavefunctions in the triplet and singlet configurations in the ratio of 3:1. For polymeric semiconductors, there is strong evidence that the triplet exciton is strongly bounded with respect to the singlet, so that 75% of the electron-hole pairs correspond to triplet excitons that do not decay radiatively with high efficiency are lost. Recent reported works has provided an estimation of the triplet concentration that is consistent with the 3:1 branching ratio expected within this simple model. However, there were also works that showed that the efficiency is higher than the 25% limit set by singlet excitons in conjugated polymer light-emitting diodes [68,69].

It is very attractive to be able to make use of the triplet exciton to get emission. One approach is to introduce species that will allow efficient triplet luminescence, *i.e.*, phosphorescence. This can be provided by high-atomic-number elements with strong spin-orbit coupling. A platinum-containing porphyrin has been successfully used as the dopant in both a molecular [70] and a polymeric host [71]. Both singlet and triplet excitons generated in the host material are collected at the porphyrin, which shows efficient phosphorescence. The devices with tris(2-phenylpyridine)iridium(III)[Ir(ppy)3] [72] as the doped material showed a very high efficiency phosphorescence emission. The energy transfer for this process is also shown in Figure 3b. As an excellent system, making full use of both the singlet and the triplet excitons, efficient white light-emitting PLEDs have progressed rapidly because polymers are easily doped or jointed with the phosphorescence materials with different color emission [73–78].

Light extraction is determined by the device structure and the refractive indices of the composed layers. A considerable portion of the light originating from emissive centers buried in a solid film never escapes due to internal reflection at the air–film interface and is scattered as edge emission or dissipated within the solid film. This is one of the major reasons why the luminous power efficiency of OLEDs remains relatively low. Although several approaches to increase the extraction efficiency have been reported, none are applicable to OLED displays.

**Figure 4.**
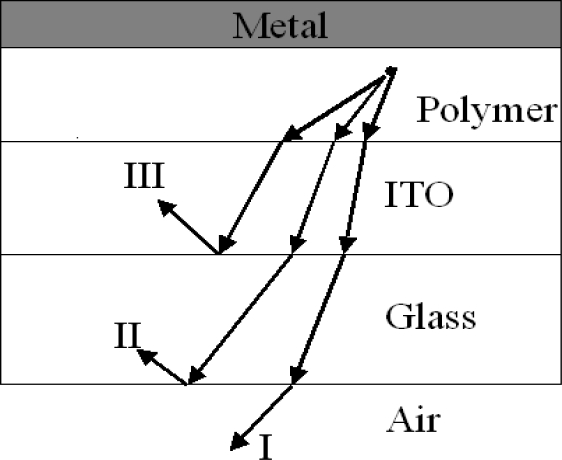
A schematic of the light-emission coupling of a PLED structure.

The typical PLED consists of a multi-layer sandwich of a planar glass substrate, a layer of ITO, a polymer medium and a reflecting cathode. The coupling problem can be easily analyzed provided that one can ignore both the microcavity effect and the diffused scattering at interfaces. If all surfaces are planar, light emitted from the back of the substrate will originate only from light emitted at angles less than the polymer-air critical angle θ_l_, given by *n*_poly_sinθ_1_ = 1 (ray I in Figure 4). Light emitted at angles larger than θ_l_ but smaller than the polymer-substrate critical angle, θ_2_, given by sin θ_2_ (*n*_poly_/*n*_sub_) = 1, is trapped in the substrate (ray II in Figure 4). Light emitted at angles larger than θ_2_ is trapped collectively in the polymer and ITO layers, and will likely be quickly absorbed by the ITO or at the cathode. It can be determined that the fraction of light escaping from the substrate (coupling efficiency), the fraction of light trapped in the substrate, and the fraction of light trapped in the polymer/ITO layers are 19, 34 and 47%, respectively, for a glass substrate and *n*_poly_ = 1.7.

It is now recognized that the coupling of electronic excitations to photon states is strongly affected by the physical structure of the diode. In device structures of the type discussed here, the presence of the metallic cathode provides a mirror which modifies the pattern of the electromagnetic modes near the cathode, setting up standing-wave states. This has the effect of reducing the radiative emission rates for polymer chains that are placed at the nodal spacings from the cathode, and in addition, there can be energy transfer into plasmon modes in the metal [79]. Studies of model structures, in which a thin semiconducting polymer layer is distanced from a metallic layer by a dielectric layer (silicon oxide), show the effects of plasmon losses at short distances (<30 nm); strong oscillations due to the standing wave states are also found, with an optimum spacing of 60 nm (dependent on the emission wavelength and the refractive index of the spacer layer) [80]. This is an important consideration for the design of efficient diodes; in this respect, heterojunction devices of the type developed [81] are particularly convenient, as the recombination process is close to the heterojunction, and this can be placed at the correct position with respect to the cathode electrode by selection of the thickness of the electron-transport layer. For diodes of the type characterized in Figure 4, where electron–hole recombination takes place over a broader region extending from the cathode, some loss of performance is inevitable. Experimentally, the optimum thickness for the polymer layer was found to be around 70 nm [82].

**Figure 5.**
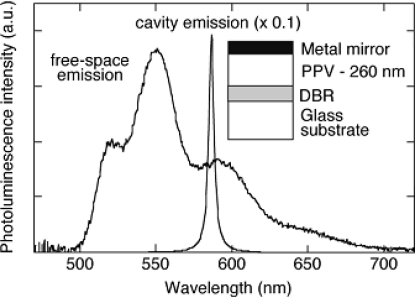
The structure and characteristic of a microcavity structure of a PLED.

The polymer LED structure is easily adapted to function as a microcavity device by the addition of a second mirror. This type of structure has been investigated with many material systems, including sublimed molecular films [83,84] and conjugated polymers [85–87]. Both metallic and dielectric (distributed Bragg reflector, DBR) mirrors have been used, as is illustrated by the structure shown in Figure 5. The Fabry–Pérot etalon formed by the two mirrors defines the allowed cavity electromagnetic modes, which have narrow linewidths for cavities formed with high-reflectivity mirrors. Emission from the excited polymer is only possible into these modes, and only when the polymer chromophore is placed away from nodes in the standing-wave electromagnetic field pattern. The spectrum shown in Figure 5 shows the very narrow emission that can be readily achieved. In addition, there is an undesirable angular dispersion of the wavelength (with a blue-shift off-axis), and a considerable redistribution of the angular dependence of the intensity. The latter may be useful when emission is required only in the forward direction; considerable intensity enhancements in this direction, as illustrated in Figure 5 for example, have been reported [84,85]. Such microcavity structures are also capable of supporting optically pumped lasing [88]. We note that considerable efforts are being made to investigate whether it is feasible to construct a polymer injection laser. In spite of the demonstration of high peak current densities (above 1000 A cm^−2^), there are considerable intrinsic problems due to excited state absorption from injected carriers [89,90].

A model based on a quantum mechanical microcavity theory has been established to compute the distribution of light emission among the three modes, and to examine the effects of the ITO thickness and the refraction index of the substrate on this distribution [91]. Finally, the modeling results are correlated with experimental measurements determined both by the far-field emission pattern and the edge emission of light trapped in the glass substrate. The coupling efficiency is found to range from 24 to 52%, which is much larger than the 19% expected from classical ray optics. The main difference between the classical and QM models arises from the relative suppression of modes at large angles from the normal by the microcavity effect.

**Figure 6.**
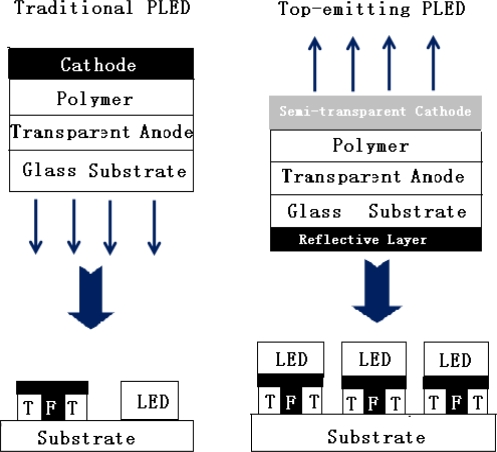
The structure and application of top-emitting PLED.

A matched light extraction and physical structure produce important effects on the characters of a top-emitting PLED. To fully exploit these advantages in displays with high information content, active-matrix drives are required, a top-emitting configuration is more suitable for active-matrix display than the bottom-emitting, *i.e.*, through-the-substrate, configuration. In this way, the PLED can be integrated monolithically on an opaque silicon substrate that also contains the thin-film-transistor driving circuit, with light emission from the top of the device. A top-emitting device can therefore make full use of the substrate area for displaying, so that the PLED can operate at lower luminescent levels with enhanced device lifetimes. For a top-emitting diode, which is shown in Figure 6, the top electrode must allow sufficient light extraction from the device, provide good charge carrier injection, and be environmentally stable. Considerable progress in the development of top-emitting organic light-emitting diodes (TOLEDs) based on the small-molecule approach, has been made in recent years. In previous work on TOLEDs, Gu *et al.* [92] employed a thin Mg-Ag layer, which was covered by an indium tin oxide (ITO) overlayer, as the semi-transparent top electrode; Hung and Tang [93] used a transparent thin film of copper phthalocynaine (CuPc), overlaid by an ITO film, as the top electrode. Although ITO has the advantage of being transparent, use of it as the top electrode is not desirable as the sputtering deposition process of ITO would cause damage to the underlying layers of the device. On the other hand, deposition of metals as the top electrode using the vacuum thermal evaporation method is relatively damage-free to the underlayers. The drawback is that, due to the high absorption coefficients of metals, the thickness of the deposited metal must be limited to a value smaller than its skin depth. Like traditional bottom-emitting diodes, balance of electrons and holes injection is crucial for high efficiency operation of a top-emitting diode. Reactive metals, such as Ca or Ba, could be used as the cathode to provide efficient electron injection. However, use of reactive metals is not practical in a top-emitting device. In a bottom-emitting device, the reactive metal could be protected by a thick overlayer of a stable metal, such as silver or aluminum. For a top-emitting device, however, the thickness of the metal overlayer is limited to the skin depth, which provides only limited environmental protection. In small-molecule based OLED, Hung *et al.* [37] have shown that a cathode consisting of a thin LiF layer and Al overlayer can provide a good electron injection property. This allows the use of environmentally stable materials as the cathode, circumventing the degradation problems inherent in cathodes made of reactive metals. This cathode configuration has been successfully applied in a TOLED with good device efficiency [94,95]. Similarly, some TPLED with environmentally stable top electrode has been achieved by modifying the electrode [96–101].

## 5. Pattering Methods for Full-Color PLED Displays

For the realization of a practical full-color PLED display technology by a cheap and effective manufacturing process, it is crucial to incorporate the three primary red, green and blue (RGB) color emissions in a pixelated array arrangement on a single substrate. Various approaches have been proposed for multi-color PLEDs. These include organic white emitters combined with pixelated RGB color filters [102] or down-conversion materials [103], and broadband emitters with pixelated microcavities [104]. Another approach involves the diffusion of dyes into the polymer by thermal or contact processes [105–107]. Printing methods such as ink-jet printing [108–110] or screen printing [111,112] are being intensively pursued for manufacturing full-color PLEDs. More recently, works have been devoted to photo-patterning approaches which can employ the low-cost spin-coating process for large area deposition to achieve high resolution display. Multi-color display was achieved by sequentially depositing and photo-patterning of red, green and blue-emitting polymers that contain photo-crosslinkable functional groups [113,114]. Another approach employs selective photobleaching of a single layer of polymer that is either doped with dye molecules [115,116] or contains blends of conjugated polymers with RGB emission colors [117]. Another method for fabricating three-color polymer light-emitting devices is by low-cost spin-coating and dry photopatterning processes employing two emissive polymer layers in tandem, each layer of which is separately patterned by a photo-oxidation process [118,119]. In this method, different combinations of the two patterned layers give rise to the three primary colors, with the emission spectrum of each color essentially being the same as that of the individual constituent red-, green-, or blue-emitting polymers, and with luminous efficiencies comparable to that of the corresponding standard single-color devices.

## 6. Conclusions and Outlook

There have been many studies on PLEDs in recent years, and progress on efficiency and stability has frequently been made in these works. The original PPV device as reported by Friend *et al.* had an efficiency of only 0.05% and a lifetime only of several hours. Now the value of the efficiency and lifetimes, which are both adequate for the requirement of most applications, are reported by several industrial companies. Table 1 shows the latest PLED performance data reported by Fyfe (Cambridge Display Technology/Sumation) [120,121].

The phosphorescence devices were given more attention recently due to their high efficiency, and being appropriate for application for the active matrix display. Many kinds of host and guest material used for polymer phosphorescence light emission have been reported in recent years and the efficiency of the phosphorescence devices is progressing very rapidly. However, it is still difficult for phosphorescence devices to maintain high efficiency over a long time.

Recently, the polymer-based white-emitting diodes have been under intense investigation and achieved significant improvement. The application of the white PLEDs is very wide, not just as the background emitters for color polymer displays, but also potentially as the backlight for traditional LCD displays. Their other potential application is for traditional lighting, to be used in various kinds of lamps in the future. The use of white PLEDs has different performance requirements very different from other single-color PLEDs used in polymer flat panel displays. In particular, they must have high luminance and stability under a high operating current density in order to meet the requirements for their application.

Compared to the development of OLED with small molecular materials, the performance of PLED is still poor in some aspects, especially the efficiency and stability of the phosphorescence and white emitting devices. Therefore, it is important that further study should be concentrated on the materials and devices to achieve higher efficiency and longer lifetime phosphorescence and in the realization of white PLEDs.

The underlying principle or mechanism in many different aspects of the PLEDs is still not fully understood. The theory and mechanism on materials and devices used in PLEDs are still attractive for scientists. The results of these works should have important scientific significant and beneficial for the development of PLEDs.

## Figures and Tables

**Table 1.** Latest PLED performance data (Autumn 2010).

**Spin/BE data@1000 cd/m^2^**	**Red**		**Green**		**Blue**	
Efficiency [cd/A]	11	31	28	50	9.0	6.0
Color (C.I.E.)	*x* = 0.67	*x* = 0.63	*x* = 0.35	*x* = 0.30	*x* = 0.14	*x* = 0.15
	*y* = 0.32	*y* = 0.37	*y* = 0.60	*y* = 0.63	*y* =0.22	*y* = 0.14
Lifetime [h]	200 k	350 k	200 k	140 k	34 k	21 k
Vd [V]	6.0	5.7	4.4	6.0	5.0	∼5.0
